# Collectanea Medica: Consisting of Anecdotes, Facts, Extracts, Illustrations, &c.

**Published:** 1822-06

**Authors:** 


### COLLECTANEA MEDICA:

CONSISTING OF

### ANECDOTES, FACTS, EXTRACTS, ILLUSTRATIONS, &c.

***Relating to the History or the Art of Medicine, and the Collateral Sciences.***

---

Floriferis, ut apes, in saltibus omnia libant,

Omnia nos, itidem, depascimur aurea dicta.

---

Art. I.—*Experiments upon Absorption.* By a Committee of the Academy of Medicine of Philadelphia. (From the Philadelphia Journal, No. 6.)

First Course.—*Upon Absorption simply.*

A. *With coloured substances.*

§ 1. THE abdomen of a healthy goat being opened, about five inches of the small intestine was filled with starch, deeply coloured with indigo, finely mixed. The lacteals were colourless, and apparently empty. After near two hours, no change was visible in them.

§ 2. The lacteals of a full-grown cat were found finely distended with a white chyle. An ounce of a strong mixture of indigo and water was thrown into a portion of small intestine, and secured there by ligatures. The lacteals immediately became colourless, and continued so for an hour and thirty-six minutes.

---

§ 3. About six inches of the small intestine of a large dog were examined, and the lacteals found colourless. It was then injected partly full of a strongly-coloured mixture of milk and indigo, and secured by tying the gut. This precaution was employed in all those instances in which the contrary is not specified. In one hour and fourteen minutes, one lacteal continued white, and all the rest were transparent. None of them exhibited any shade of colour.

§ 4. In the goat mentioned in § 1, about five inches of the great, and an equal portion of the small, intestine, were filled with milk; the lacteals being of the same transparency as above described.

These vessels continued, for a considerable time, to present the same appearance, after the animal was killed.

§ 5. A sheep, apparently healthy, was opened, and milk introduced into three different portions of intestine. In the upper parts of these, or those nearer the stomach, lacteal vessels containing chyle were seen intermixed with others which were transparent.

A white fluid was, in all instances, seen to be contained in a few of the lacteals on the mesentery, and in several of those on the gut, for the space of a few minutes: it generally soon disappeared.

§ 6. In a full-grown female cat, a quantity of milk was thrown into a portion of small intestine, of which the lacteals were colourless. They continued colourless.

§ 7. In a large dog, a portion of small intestine, near the stomach, was partly distended with warm milk. The lacteals were white and transparent, mixed. They all soon became clear, and continued so till the termination of the experiment, in fifty-three minutes.

§ 8. In a full-grown female cat, the lacteals were found filled with white chyle. An ounce of an infusion of powdered rhubarb was thrown into a portion of the small intestine, together with the powder. The lacteals immediately became transparent, but colourless, and continued so till the end of the experiment, in one hour and forty-five minutes.

§ 9. In a female cat, the lacteals were found somewhat distended with white chyle. A quantity of strongly-coloured infusion of rhubarb was thrown into the small intestine, and the part tied and returned into the abdomen. In other instances, the portions of intestine on which we operated were sometimes returned into the cavity of the abdomen, sometimes covered with warm cloths, and sometimes not well protected from the cooling effect of the atmosphere. The experiments were generally tried at a time when the thermometer ranged 70° to 85°.

The lacteals, in this case, became colourless and almost perfectly transparent. On puncturing them, they emitted abundance of a fluid resembling chyle and water.

§ 10. In a large dog, the lacteals of a portion of small intestine were found white and colourless, mixed. The animal continuing vigorous, strongly-coloured infusion of rhubarb was thrown into the segment of intestine warm. The lacteals continued for some time apparently in the same state, and underwent no change of colour.

Some white vessels were visible in fifty-four minutes. In this instance, as in many others, the intestine was not emptied of its contents. This was done whenever the part appeared occupied with more fluid than necessarily adheres to it.

We believe that there was a sufficient quantity present in this case to afford some chyle.

§ 11. In a female cat, the whole of whose lacteals were white and distended, highly-coloured infusion of alkanet and cochineal, to the amount of half an ounce, was thrown into a portion of small intestine. The part was then returned into the abdomen. After a considerable interval, (about an hour,) the lacteals were uniformly transparent and colourless, and afforded a clear fluid on puncture.

§ 12. In a large dog, the same employed in § 3, (and who had become faint and weak by previous experiments,) a quantity of infusion of cochineal, the colour of which had been rendered more intense, and converted to purple, by the admixture of pearl-ash, was introduced into the small intestine. The animal made efforts to vomit. The lacteals, which in this instance were originally transparent, continued so, and did not assume the slightest colour.

§ 13. In a female cat, whose lacteals were uniformly white, and moderately distended with chyle, strongly-coloured infusion of madder was thrown into the small intestine, and the part returned into the abdomen. After a considerable interval, these vessels were found colourless, and afforded, on puncture, a considerable stream of watery fluid.

§ 14. The last experiment was repeated on a large dog, whose lacteals were colourless. They continued so.

§ 15. The same experiment was repeated on a ram, near his stomach. The lacteals were originally filled with white chyle: in nineteen minutes they were equally so; in fifty-six minutes, some were transparent, others faintly white, but all free from colour.

B. *With odoriferous substances.*

The remaining experiments were all comparative, between the lacteals and blood-vessels.

§ 16. A portion of the small intestines of a ram, whose lacteals were generally filled with white chyle, was secured, and a quantity of alcoholic solution of camphor was thrown in. In five minutes many more white lacteals became visible, and all appeared more distended than in the beginning of the experiment. In nine minutes the intestine was much less distended, much fluid having been removed. In eleven minutes all the lacteals gradually became nearly transparent. In eighteen minutes lymph, of watery appearance, was obtained from lacteals, and no smell of camphor was afforded, although all the parts smelled strongly of it. Camphor, in considerable quantity, remained in the intestine. The parts subjected to this experiment assumed and retained a bright red colour, apparently from the stimulus employed. In thirty-one minutes from the injection of camphor, blood was obtained from a mesenteric vein, which had no smell of camphor.

§ 17. Tincture of camphor was carefully introduced into the small intestine of a kitten, and secured. In thirty-two minutes, blood, from a large mesenteric vein of the part, had no smell of camphor. In fiftyeight minutes blood from the jugular vein, and fluid from the thoracic duct, had no smell of camphor.

§ 18. Tincture of camphor and mint wate., to the amount of about half an ounce of each, had been thrown into the small intestines of a cat, in two places, and secured by tying the gut; also tincture of camphor into the great intestine of the same animal. In an hour and a quarter after the commencement of the experiment, the blood of a mesenteric vein had no smell of either article.

§ 19. In a terrier female, half an ounce of tincture of camphor, with as much water, were injected into the stomach. The effects of this article on the nervous system were immediately apparent in a high degree. From two and a half to three minutes, the animal was in violent convulsions; then vomited freely, 'and in thirteen minutes was pretty well recovered. Half an ounce of tincture of camphor was then injected *per anum*, mixed with an ounce of water. She soon discharged a considerable portion of the mixture; but in four minutes' time the effects of camphor on her nervous system were very apparent. In twenty-seven minutes and a half she was killed by a blow on the occiput.

Tincture of camphor was found in the stomach, on opening it after the conclusion of the experiments. In thirty minutes, blood from the inferior mesenteric vein did not smell of camphor. In forty minutes, lymph and chyle, mixed, from the thoracic duct, did not smell of camphor.

Sections of the liver did not smell of camphor, which would have been conveyed there, if absorbed by the veins.

§ 20. A cat had escaped, and lived three days in a place without food, owing to our not being able to discover her. Her lacteals were, of course, in a state of great exhaustion. Tincture of camphor and water, mixed, were injected into the stomach; and, within twenty-five minutes, two clysters of the same were given. She was much intoxicated; when, in twenty-nine minutes, she was killed by a blow on the occiput. In thirty-four minutes, a pellucid fluid, from a lacteal, did not smell of camphor. In thirty-six minutes, lymph, with some chyle, from the thoracic duct, did not smell of camphor. In thirty-nine minutes, blood from the vena portarum did not smell of camphor. In forty minutes, sections of the liver did not afford the smell.

§ 21. Several pieces of camphor were introduced into the cellular substance of the thigh of a dog. Tincture of camphor and water were then injected into the abdominal cavity, into the stomach, and into the rectum; the latter being performed twice. In forty-one minutes, the dog was killed by a blow on the occiput. In forty-six minutes, blood from the right auricle of the heart had no smell of camphor. In fifty-six minutes, large quantities of blood had no such smell.

Lymph from the thoracic duct, and sections of the liver, had no smell of camphor. The smell had nearly disappeared in the abdominal cavity, where it had been injected: a very singular phenomenon, indeed, as this smell was not to be found in any part of the fluids or tissues of the animal.

The tincture was greatly diminished in the rectum; as considerably less was found there than had been retained. The breath was carefully smelled, but no camphorated scent discoverable.

In order to ascertain whether our senses were in a proper state for this investigation, we mixed a small quantity of tincture of camphor with some blood, separately from the animal, and smelled it: no difficulty was found in distinguishing the usual sensations.

§ 22. An ounce of tincture of camphor, diluted with water, was introduced into the abdomen of a cat; which became instantly insensible. In twenty-five minutes, the left cavity of the pleura exhaled a smell of camphor. The blood of the heart smelled of the drug, but less strongly. The fluid of the thoracic duct afforded no camphorated smell.

§ 23. In a dog, between two and three months old, about two ounces of strong camphorated spirits were injected into the cavity of the abdomen, and the wound sewed. In five minutes, the effect of the drug existed in a high degree, and the animal became insensible. In ten minutes, the mouth and breath smelled of camphor; and, in twenty minutes, strongly so. Blood was obtained, in ten minutes, from the right jugular vein; in twenty minutes, from the left jugular; and, in forty minutes, from the transverse vein of the neck : but in none of these instances was the least camphorated or spirituous smell discoverable.

The cavity of the pleura was next opened, and afforded no smell of the substances injected.

In forty-five minutes, one-fourth of a drachm of chyle was collected, but it afforded no smell.

The ventricles of the brain afforded no camphorated nor spirituous smell.

The thoracic duct became strongly distended again, after being evacuated in part, not less than twenty-five minutes after the death of the animal functions.

§ 24. An ounce of tincture of assafœtida, with an equal quantity of water, was injected into the abdomen of a cat. The breath smelled very strongly of assafœtida in three minutes; the animal was intoxicated within the same time.

In ten minutes, blood was obtained by cutting the animal's throat; and slightly, but distinctly, emitted a peculiar smell, which we compared to assafœtida, but which was modified.

The serous cavities smelled of both assafœtida and spirits, but principally of the latter. The mucous, on the contrary, smelled of both, but principally of the former. The chyle in the thoracic duct emitted the same peculiar smell with the blood. The muscles gave no smell of the drug; the urine a powerful one.

§ 25. A small dog was compelled to swallow half an ounce of tincture of assafœtida, diluted with water. The animal soon became evidently affected by the drug. Blood was obtained from a vein of the stomach in little more than thirty-two minutes, and had no smell of assafœtida. The cavities of the pleura and pericardium were found devoid of the fœtid smell. In fifty-six minutes, chyle and lymph from the thoracic duct did not smell of the drug. Blood from the carotid artery did not afford the smell in fifty minutes.

The breath here, of course, was imbued with the odour in the mouth and œsophagus. It is proper to remark, that this animal vomited at twenty-eight minutes, but that much of the article used was found in his stomach after death.

§ 26. An ounce of tincture of assafœtida was thrown into the rectum of a large cat, mixed with an ounce of strong solution of prussiate of potass. The rectum was then secured with a ligature.

In four minutes, the smell of alcohol was perceived in the breath; and, in twenty-three minutes, a smell of assafœtida had gradually followed. In thirty-three minutes, blood from the carotid artery and jugular vein, mixed, smelled of assafœtida and alcohol. In forty-four minutes, the abdominal cavity smelled distinctly of both these articles; in forty-six minutes, the urine did not smell of them.

The phenomena afforded by the presence of prussiate of potass fall under another head.

C. *With chemical substances.*

§ 27. A cat was compelled to swallow one and a half grains of prussiate or ferro-cyanate of potass, dissolved in two ounces of water: a solution, of which half a drop, mixed with two ounces of fresh water, struck a deep blue, on the addition of sulphate of iron. The animal was killed in twenty-seven minutes, by a blow on the occiput. Blood was obtained from the heart, and tested with sulphate of iron: no appearance of the prussiate was discoverable in sixty-four minutes. An infusion of sections of the kidneys was tested in the same manner, but without discovering the prussiate.

No fluids could, in this instance, be obtained from the thoracic duct or from the bladder.

§ 28. A large healthy cat was procured; the abdomen opened, and three ounces of solution of prussiate of potass, containing half a drachm of the salt, were injected and secured. In thirty-five minutes, it was killed by a blow on the occiput, and blood procured from the jugular vein and heart, as soon as possible after securing the thoracic duct: the ligature included the transverse vein of the neck. The serum of the blood from the heart afforded a light blue, when tested with sulphate of iron and water: that from the jugular vein was tested in the same way, when, "if it changed to blue, it was scarcely evident." Half a drachm of the semi-transparent contents of the thoracic duct was mixed with a drachm of water; when one drop of a solution of sulphate of iron being added to it, afforded a beautiful blue colour.

After an interval, these experiments were repeated without the addition of water, and while the chyle, freshly obtained for the purpose, was still fluid. The colour obtained both from the contents of the jugular vein and heart was deep, and from the chyle intensely so.

§ 29. A large healthy cat was opened, and two ounces and a half of the solution of prussiate of potass injected into the abdomen. The serum of the blood and the chyle afforded results exactly similar to those in the last experiment. The fluids were arrested in their vessels, for the purpose of being extracted, at thirty-five minutes.

§ 30. The same experiment was repeated on a half-grown kitten, after previously tying the ductus thoracicus. An ounce and a half of solution was injected. But here it is proper to state, that a considerable quantity of the solution was effused into the cellular structure of the abdominal integuments. The kitten was killed in thirty-five minutes, and blood obtained from the jugular vein: this afforded a light blue, on application of the test. The milky fluid from the thoracic duct afforded a strong blue; the urine afforded a light blue.

§ 31. In order to satisfy ourselves that these phenomena arose from the presence of the extraneous body in the vessels, serum of blood, and lymph and chyle from the thoracic duct, were produced, under similar circumstances, from a cat, and tested with the sulphate of iron. No change, however, was produced; thus evincing that the test was not fallacious.

§ 32. An ounce of solution of sulphate of iron was injected into the peritoneum of a kitten. No indication of this substance was afforded, in thirty-two minutes, by the test of prussiate of potass applied to the serum.

This was repeated by one of our number, with the effect of producing a greenish tinge approaching to blue, probably resulting from the colour of the sulphate itself; which, however, soon subsided into a thick mucous sediment, leaving the supernatant fluid clear.

§ 33. The experiments described in § 29 and 30 were repeated, with results so precisely similar, that it was not thought necessary to describe them more minutely.

§ 34. A quantity, not precisely determined, of solution of prussiate of potass was injected into the stomach of a large cat, by the mouth. In forty-five minutes, the test faintly, but certainly, indicated the presence of the prussiate in the chylous fluid of the ductus thoracicus. The urine indicated it strongly, but the clear serum of the blood gave no appearance of it at all. It is right to mention that, in this instance, the œsophagus being cut, it is within the bounds of possibility that a small particle of dissolved prussiate may have thus found its way into the vessel that collected the chyle, although we believe it was not so.

The stomach was found perfectly empty.

§ 35. Two ounces of solution of prussiate of potass were injected into the abdomen of a cat; and, in about twenty-five minutes afterwards, about one drachm of the solution of sulphate of iron was thrown into the right jugular vein. In two or three minutes, the animal was convulsed violently, and part of the solution expelled from the abdomen.

In thirty-five minutes from the first injection, the ferro-cyanate was detected in the contents of the thoracic duct.

The serum of the blood was transparent, and did not indicate the ferro-cyanate.

§ 36. A half-grown cat was made to swallow an ounce of the solution of prussian alkali. In two minutes she was bled to death, and the prussiate clearly indicated in the urine, but not, in the least visible degree, in either the chyle or the serum of the blood.

§ 37. A large male cat was forced to swallow near two ounces of solution of prussiate of potass. In thirty-five minutes, urine, serum of blood, and fluid from the thoracic duct, were collected; neither of which indicated the presence of the salt in question, in the slightest degree. Notwithstanding this, a great portion of the solution had been removed from the stomach by animal processes, as not more than a drachm remained in that viscus after death.

§ 38. Another large cat was made to swallow two ounces of the prussic solution; and, in forty-five minutes, the fluids were tested in the usual manner, but without discovering any absorption of this chemical agent.

§ 39. Two ounces and a half of the solution were injected into the peritoneal cavity of a young cat. In twenty-six minutes it was detected in the urine, and in the serum of the blood. No chyle was collected.

§ 40. A middle-sized cat was forced to swallow about two ounces of prussiate of potass. She first became very sick, then recovered, after having a fluid stool, and afterwards slept; but was still unable to walk steadily. In two hours, twenty-five minutes, she was killed. The urine indicated the salt; the chyle did so but faintly. It was not evident to satisfaction in the serum of the blood.

§ 41. Two ounces of the prussiate solution were injected into the abdomen of a kitten; which was killed in ten minutes, and the fluids collected as soon as possible. On applying the test, the urine did not contain a discoverable quantity of the prussian alkali, and the serum of the blood not so as to be evident in a satisfactory manner; while the fluid of the thoracic duct exhibited it slightly, but distinctly.

§ 42. A small cat was made to swallow an ounce of the solution; and, ten minutes after, another ounce was thrown into the rectum. In an hour the cat was killed. No sign of the prussiate was found in the serum of blood from either the heart or the mesenteric vein, or in the urine. The chyle was lost.

§ 43. A large cat, mentioned in article 26th, was compelled to retain an ounce of the prussiate solution, mixed with an ounce of the tincture of assafœtida, by means of a ligature on the anus. In thirty-three minutes the animal was killed, and the fluids prepared for examination within thirteen minutes after. Prussiated potass was found in the urine, forming deep blue with sulphate of iron, acidulated with nitric and muriatic acids. Chylous fluid, from the thoracic duct, afforded a distinct greenish blue with sulphate of iron and nitric acid. Serum of blood, both arterial and venous, from the neck, separately, afforded a green with sulphate of iron and nitric acid.

To discover how much of the colour in the serum was owing to iron, which the muriatic acid contained, this fluid was tried, first with nitric acid, which gave a white albuminous precipitate; secondly, with muriatic acid in addition, which gave a slight green; when, thirdly, sulphate of iron was added, which gave a more intense green, tending to blue.

§ 44. An ounce and one half of solution of the prussiate was injected, with much pains, into each thigh of a large cat, and the wound stitched. At one hour and twenty minutes afterwards he was killed. The urine and chyle very strongly indicated the presence of the salt; the serum of the blood exhibited it slightly. It was discovered in the liquor pericardii.

§ 45. A small kitten was placed in a bath composed of the prussian solution above mentioned; and, after thirty-five minutes, it was removed and killed. No indication of the salt could be obtained, though examined in the usual manner.

§ 46. Two ounces of a solution of chromate of potass were injected into the peritoneal cavity of a large cat. In thirty-five minutes she was killed, and the fluids tested, as soon as compatible with accuracy, by means of the subacetate of lead. In neither urine, fluid of the thoracic duct, nor serum of the blood, was the chromate detected.

D. *With Poisonous Substances.*

§ 47. In a kitten, two months old, the capsule of Glisson was included within a tight ligature, so as to put an end to all circulation in the blood-vessels which traversed it. Two-thirds of an ounce of nux vomica had been infused in eight ounces of boiling water, and with this several inches of small intestine were distended. The animal soon began quick respirations, with protrusions of the tongue, which continued nearly till her death. In fourteen minutes violent spasms took place. In seventeen more, violent spasms, distinctly tetanic, with opisthotonos. In twenty-three minutes the animal expired, after repeated tetanic spasms.

§ 50. A kitten, about one month old, was opened, and the capsule of Glisson tied in the same manner as in the last experiment, in order to ascertain how far this violence affected the results above described.

More frequent breathing took place, together with protrusion of the tongue. In seventeen minutes, appeared to breathe tolerably. In twenty-two minutes,—has been for some time dying, with slow respiration, progressively declining. In thirty minutes, ceased to struggle.

No tetanic symptoms, and no spasms whatever, were observed in this animal. The heart, which was first examined in this case, continued to palpitate for nearly an hour after death, and was easily excited again to action, after it had ceased, by puncturing it with a needle.

§ 51. A full-grown terrier female, that had had young, and appeared to be in good health, was procured. The lacteals were distended with chyle. The capsule of Glisson was tied, with all its contents, and an ounce of the above-mentioned infusion of nux vomica injected into the small intestine, and secured. The animal died in twenty-eight minutes, with the pupils of the eyes dilated, the corners glassy, and with well characterized tetanus.

§ 52. As a few drops of the decoction were spilled in both instances, and came in contact with the peritoneum, we made the following trial of the effect of this substance on that membrane. The intestines of a cat, weakened by several preceding experiments, were wet, on their peritoneal coat, with the infusion above mentioned. No effect was produced in seventeen minutes. The animal lived a longer time than could have been expected from the violence done her, and died without tetanus or other symptoms of the poison.

§ 53. A cat, on which several of the above-mentioned experiments with coloured substances had been tried, still retained considerable vigour; when the mesenteric veins of a portion of intestine, four or five inches long, were secured, and the gut distended with the decoction of nux vomica. Death ensued in eleven minutes, although the animal had borne other experiments for an hour and twelve minutes. We are, however, not prepared to say that this experiment was entirely satisfactory, as its results depended on circumstances which could only be estimated by our judgment, and could not be brought under the test of precise mensuration.

§ 54. A portion of small intestine, about two inches in length, in a kitten, was separated from the remainder by cutting it off; the arteries and veins, where visible, were then secured, including a small vessel which appeared in about two minutes after the commencement of the experiment. Half an ounce of tincture of nux vomica was then injected. In seventeen minutes no effect was produced.

This experiment, although mentioned here for the sake of candour, was far from being circumstanced so as to produce reliance upon deductions drawn from it; as several lacteals, and most, if not all, of the nerves, were evidently included within our ligatures, and the part was, very probably, early deprived of vitality.

Not having it in our power to obtain either of the species of upas, we thought it proper to make some trials with prussic acid; although we acknowledge that, to our minds, the supposition appears absurd, that an agent, whose effect is sometimes so instantaneous, could require for its operation the circuitous route of absorption.

The prussic acid which we employed was the alcoholic solution recommended by Dr. Thomas Cooper, much weakened, and was tried in the following manner.—One drop was placed first on the eye, and then on the tongue, of a young kitten, with impunity. Half a teaspoonful was then injected into the stomach; and death, with the usual symptoms, followed in one minute. As the acid had been kept for some time previously to this trial, and was carefully put away, and used within three days, it is presumed no very great alteration in its strength took place.

§ 55. The thoracic duct and vena portarum of a kitten, two months old, were tied. The lacteals were white, and very conspicuous. About one-fourth of a drachm of the prussic solution was introduced into the small intestine, and the orifice tied. The kitten died in seven minutes, with screaming and struggles, but with no tetanic symptoms. In twenty-four minutes the heart was examined, and found perfectly motionless, and not susceptible of excitement by puncturing. On opening the intestine operated on, the mucus which had come in contact with the poison was stained reddish brown. The first inch of the bowel was opaque and deprived of its peristaltic motion, although the rest manifested it in a high degree.

§ 56. The vena portarum of a half-grown kitten was tied, and nearly a drachm of alcoholic solution of prussic acid was injected into the small intestine and secured by a ligature above and below. This experiment is inserted for the sake of candour, although we consider the prussic acid much weakened. The lacteals, which were completely distended with chyle, became empty, as if paralyzed. In fourteen minutes a rigid spasm was observed. In fifteen minutes and a quarter the animal, which had been lying quiet, with the last-mentioned exception, and apparently gradually losing strength, expired.

Second Course of Experiments *on the Question, whether Infiltration can take place through living Tissues, in direction transverse to their Texture.*

A. *With coloured and chemical substances.*

§ 57. Ink was confined upon two parts of the mesentery of a living cat. After a considerable interval, the opposite side of this membrane was examined with care, and no mark of infiltration was found.

§ 58. More than a drachm of ink was poured into the right pleura of a living female cat, and suffered to remain there nine minutes, including seven of apparent death, but during which the organic life must have continued in the part. The membrane was then carefully dissected from considerable surfaces both of the lungs and the thoracic parietes, but no coloured infiltration was discovered.

§ 59. Two ounces of ink were injected into the peritoneum of a cat. In thirty-five minutes the animal was discovered to be dead. After previously examining another part, the urine was obtained, and the bladder examined. The bladder, whose outer surface was entirely black, from having had nearly the whole of the ink in contact with it, owing to the position of the animal, did not show the least mark of infiltration on its inner surface; nor did the urine either evince its admixture or the presence of iron alone, as exhibited by the addition of ferro-cyanate of potass. No other part was, in this instance, examined for infiltration.

§ 60. An inch and a half of the right internal jugular vein of a ram were uncovered on the side which lay uppermost, and for one half of its circumference; leaving, however, the natural attachments of the remaining half of the vein, in order to avoid risking the destruction of the vitality of the part. Muriatic acid, mixed with water in the proportion of two drachms to eight ounces, was kept constantly applied to the part. In a few minutes the part exposed became quite black; and in ten minutes the whole section of vein, included, as it had been from the beginning of the experiment, between two ligatures, was removed and washed. The cavity was then exposed, and the blood and inner surface of the vein applied to our tongues, without the perception of any acid taste.

B. *With odoriferous substances.*

§ 61. A ligature was made round the small intestine of a kitten, near the stomach, and tincture of camphor introduced. The orifice was then tied, and all the parts which had been in contact with the injecting instrument carefully cut off, so that not the least smell of the drug was any longer perceived. In one minute a smell was perceived, but we could not distinctly characterize it as that of camphor. In fourteen minutes, camphor was distinctly smelled from the outside of the intestine. In fifty-eight minutes this smell was stronger. The circulation had gone on for all this time, and clear lymph had continued to traverse the lacteals.

§ 62. A small vial of tincture of camphor, corked, and externally totally devoid of smell, was introduced into the large intestine of a cat. The gut was then carefully tied, and the cork pressed out of the neck of the vial, without disturbing the ligature. In fifteen minutes no smell was perceived ; but, after some time, that of camphor was very obvious.

§ 63. Tincture of camphor was then introduced into the small intestine of the same cat, and secured between two ligatures; no vial being obtained sufficiently small to enter this part. The intestine had at first a faint smell of camphor, which disappeared in three minutes. In three-quarters of an hour, a slight smell of this drug was discovered, and in one hour and twenty minutes it became quite distinct and evident; the part during all this time retaining its life and vital actions.

§ 64. A terrier female had half an ounce of tincture of camphor, mixed with as much water, injected into the stomach. In twenty-seven and a half minutes she was killed ; and, other observations having been made on her, which occupied the time as far as forty-two minutes, the stomach was examined. It did not smell of camphor, although this substance was immediately afterwards found in the cavity.

§ 65. Mint-water was thrown into the small intestine of the cat employed in the last-mentioned experiment but one, and secured so that no smell was perceptible. In about fifteen minutes the smell of mint was perceived, and in forty-four minutes it was very apparent, although the actions of the part continued during the interval.

§ 66. A small dog was compelled to swallow half an ounce of tincture of assafœtida. In twenty-eight minutes the animal vomited, being at the same time much under the influence of the drug. No smell could be perceived on the outside of the stomach. In fifty minutes the carotid artery was divided. In seventy-seven minutes the smell could not be perceived on the outer surface of the stomach. In four hours twenty minutes it was indistinct, although the stomach was separated from the body, and partially dried. On opening this viscus, however, the smell was very strong.

---

Art. II.—*On the Use of the dilute Sulphuric Acid in Cutaneous Affections.* By J. Fosbroke, Berkeley.

My experiments with the dilute sulphuric acid, in *large doses,* have been continued with success.—A lady, who had been long subject to very distressing chronic disorder of the digestive organs, attended with temporary congestions of the spleen, became affected with the *lichen agrius* on the back of her hands and inside of her thighs. It proved very obstinate in its resistance to several topical plans of treatment. I prescribed the acidum sulphuricum dilutum in a decoction of the dermis of the elm, and of the wood of the garden nightshade *(solanum dulcamara)*; using at the same time, as a wash, a decoction of the deadly nightshade *(atropa belladonna)*. She literally told me that irritation was immediately "deadened." The wash stimulated the diseased surface very much. It was applied previously to the use of the acid, and continued afterwards. The acid can rarely be taken, "in spite of the teeth," long; but the inconvenience may be obviated by using a quill as a syphon, projecting beyond the teeth towards the fauces. Dr. Willan has recommended the acid in infusion of roses, with bark, in this disease; but his dose is not large, on which all specific effect depends.

Mr. Turner, a veterinary surgeon, in large practice at Wotton-under-Edge, Gloucestershire, has communicated to me the two following cases of the use of dilute sulphuric acid in skin diseases.—A man suffered severely in consequence of taking violent exercise, by which he became heated, and perniciously drinking cold water immediately afterwards. Small blotches, red, and level with the cuticle, appeared spontaneously. He took the dilute sulphuric acid, beginning with six or seven drops, and continued it for three months; during six weeks of which time the dose consisted of 160 gtt. of the acid undiluted, taken more than once a-day. It effected a complete recovery of his general health, as well as local disorder.

A man who had had, during a long time, swollen extremities and erratic rheumatism, especially affecting the bronchia, recovered by the use of this acid in large doses. He now enjoys robust strength; and his respiration is so much amended, that his capability for psalm-singing, to which he was much addicted, is in higher perfection than it has been for many years past.

I conceive that our practice must err, if we do not regard, with restorative intentions, the condition of the grand system of secretion; and, though I greatly rely upon the alterative influence of sulphuric acid alone, it may be well, in most cases, to treat the liver as an agent ever to be suspected in disordered cutaneous actions. For this, let there be given Plummer's pill, in purgative doses; or the pil. cambog. comp. ℈ij., pulv. antim. ℈j. fiant. pil. l. xii., a medicine of no mean powers. At the same time, by this plan we not only confine our curative intentions to the disease apparent, but add into our general schemes of treatment the original disorder of the internal organ or organs; for all that I have seen concurs, for the most part, with the maxims of Dr. Jenner, who, in an ingenious unpublished treatise on the pathology of the skin, announces the probability that "the design of nature, in making eruptions on the external surface, is to protect vital organs, by transferring diseased action to parts not vital."

P.S.—I have, since the publication of my former paper, been able to retrace the literary information which suggested to me the use of dilute sulphuric acid in carbuncle. It occurs in the Edinburgh Medical Commentaries. The quotation may gratify those who may have never read them:

"We are informed, by Dr. Gahn, that the vitriolic acid has of late been much, and successfully, employed, in some places of Germany, in the cure of the itch. It was first used for this purpose in the Prussian army, in the year 1756, by Dr. Cothenius, first physician in Prussia. This practice was afterwards described in a treatise, entitled "Dissertatio de Olei Vitrioli usu in quibusdam Scabiei speciebus," written by Dr. Helmich, and printed at Hale in 1762. It was also described by Dr. Baldinger, in his Treatise on the Diseases of the Army, which is written in the German language. Dr. Schroeder, professor of physic at Gottingen, informed Dr. Gahn that he had frequently employed it in practice; and that he never failed to cure the itch by means of it, in fourteen days at farthest."*

Dr. Duncan, senior, also observes,† "I have thoughts of putting this patient on the use of the vitriolic acid; a medicine which has of late been particularly recommended for the cure of itch: and I am told that the efficacy of it has been fully confirmed, both in cases of scabies sicca and humida, by the able physician Dr. Baldinger, of Gottingen. And if, after the failure of the present plan, it shall succeed in the instance before us, I shall have a higher opinion of it than ever. And, if the vitriolic acid fail from internal use, I have thoughts of trying it externally; for, when conjoined with hog's lard, it forms a very elegant ointment."

Sydenham, almost the most delightful, as well as correct, of our medical classical writers, speaks with great warmth of the efficacy of sulphuric acid in checking the increase and destructive progress of confluent small-pox pustules, and, consequently, of the fever which they excite.

The external application of the deadly nightshade was formerly tried, and well spoken of, by Dr. Graham,‡ who used it in cases of ill-conditioned tumors and ulcers. Of this I was not aware when my own experiment was made.

*(Edinburgh Medical Journal.)*

---

* *Edinburgh Medical Commentaries*, pp. 103-104.

† Dr. ANDREW DUNCAN's *Medical Cases*, vol. i. p. 48.

‡ *Edinburgh Medical Commentaries*, vol. i. p. 419.

